# Impact of Chronic Suppurative Otitis Media on Quality of Life and Psychological Well-Being: A Cross-Sectional Study

**DOI:** 10.7759/cureus.54150

**Published:** 2024-02-13

**Authors:** Shaila Sidam, Anjan K Sahoo, Utkal P Mishra, Vikas Gupta, Anuradha Kushwah, Prasanta K Sahoo

**Affiliations:** 1 Otolaryngology - Head and Neck Surgery, All India Institute of Medical Sciences, Bhopal, Bhopal, IND; 2 Psychology, All India Institute of Medical Sciences, Bhopal, Bhopal, IND

**Keywords:** quality of life, psychosocial, psychological, mental, depression anxiety stress scale, depression, chronic suppurative otitis media, anxiety

## Abstract

Background: Chronic suppurative otitis media (CSOM) is a prevalent and persistent middle ear condition that not only affects auditory health but also potentially influences various aspects of an individual's life. This study explores the correlation between CSOM, depression, anxiety, and stress, using the 21-item Depression, Anxiety, and Stress Scale (DASS 21), also assessing quality of life (QoL) using the Chronic Ear Survey (CES) questionnaire. The primary objective of this study was to gather prospective audiological data along with information on both disease-specific quality of life and psychological well-being, utilizing validated measurement instruments.

Methods: This cross-sectional study was conducted at a tertiary care center in Central India, involving 182 patients with CSOM. The study included individuals aged 18 years and above diagnosed with CSOM in at least one ear. Patients with a history of psychological disorders, head injury, and those with comorbidities such as diabetes, hypertension, and chronic heart diseases were excluded. Pure tone audiometry was employed for hearing evaluation, while the assessment of psychological well-being utilized the DASS 21 questionnaire. Furthermore, the quality of life was evaluated using the CES tool.

Results: From the initial cohort of 182 patients diagnosed with CSOM, 32 were excluded based on predefined criteria, resulting in a final sample of 150 patients. The cohort, with a mean age of 34.3 years, exhibited a predominantly female population (63.3%). Psychological assessments using DASS 21 revealed depression in 22 (14.7%) patients and anxiety in 23 (15.3%) patients. Among those with depression, majority of the participants had mild depression. Similarly, among those with anxiety, the majority were found to be experiencing mild anxiety. Bilateral CSOM demonstrated a higher prevalence of anxiety and depression, establishing a significant association. QoL parameters, assessed by the Chronic Ear Survey, indicated a more adverse impact in bilateral cases across all categories except symptoms. Correlation analysis between psychological well-being, quality of life, and hearing loss severity yielded statistically significant results.

Conclusion: CSOM with the symptom of hearing loss can lead to reduced QoL and psychological well-being in the affected individuals. This study highlights the psychological impact of CSOM, particularly in bilateral cases and severe hearing loss. Integrating psychological support into treatment plans is crucial for comprehensive patient care. Regular assessments are essential for guiding timely interventions, ensuring a holistic approach to enhance both quality of life and psychological well-being in individuals affected by CSOM.

## Introduction

Chronic suppurative otitis media (CSOM) represents a persistent and prevalent middle ear pathology, characterized by chronic inflammation of middle ear leading to recurrent ear discharge and impairment of hearing. Beyond its physiological implications, CSOM can significantly influence various aspects of an individual's life, extending beyond the realm of auditory health. According to the latest data from the World Health Organization (WHO), an astounding 65-330 million individuals worldwide are suffering from discharging ears, and within this population, a substantial 60% are experiencing significant hearing impairment [1]. The spectrum of this hearing impairment spans from partial to complete loss of auditory function, categorized as mild, moderate, severe, and profound. This auditory challenge not only hampers communication skills, hindering social interactions and professional endeavors, but also exerts a profound impact on mental health, resulting in diminished self-perception, happiness, and overall psychosocial well-being. Profound hearing impairment, in particular, can give rise to social isolation and depression [2]. The WHO characterizes quality of life (QoL) as an individual's subjective evaluation of their position in life, considering personal goals, expectations, patterns, and concerns within the cultural and value framework to which they belong [3]. In the context of CSOM, various factors such as socioeconomic status, duration of the disease, and occupational stress not only influence typical audiologic changes seen in patients but also play a role in the manifestation of psychological distress [4]. As a condition with both physical and psychosocial dimensions, understanding the impact of CSOM on the quality of life and psychological well-being of affected individuals is essential.

To date, a very limited number of studies have been undertaken in the Indian subcontinent to evaluate the impact and correlation of CSOM on the quality of life and psychosocial well-being of the affected individuals. The primary objective of this study was to explore the correlation between depression, anxiety, and stress in individuals with CSOM, utilizing the 21-item Depression, Anxiety, and Stress Scale (DASS 21) questionnaire. Additionally, we evaluated the QoL parameters in CSOM patients using the Chronic Ear Survey (CES) questionnaire.

## Materials and methods

This was an institution-based cross-sectional study that was conducted in the Department of Otorhinolaryngology - Head and Neck Surgery of a tertiary care referral center in Central India. This study was reviewed and approved by the Institutional Human Ethics Committee, AIIMS, Bhopal, India, before the commencement of the study. Written and informed consent were obtained from all study participants. Patients over 18 years of age diagnosed with chronic suppurative otitis media in at least one ear were included in the study. Patients with a history of psychological disorders, head injury, and those with comorbidities such as diabetes, hypertension, and chronic heart diseases were excluded from the study. A detailed overview of the comprehensive inclusion and exclusion criteria is presented in Table 1.

**Table 1:** Inclusion and exclusion criteria

	Criteria
Inclusion criteria	Patients over 18 years of age diagnosed with chronic suppurative otitis media
Exclusion criteria	Patients with a history of psychological disorders or head injury; patients with comorbidities such as diabetes, hypertension, chronic heart diseases, tuberculosis or malignancy; those with intracranial complications of chronic suppurative otitis media

A detailed history was obtained, encompassing details such as the onset, duration, progression of ear discharge, and nature of the treatment received for CSOM. Additionally, any complications associated with CSOM were documented. Clinical assessments included the determination of disease laterality (unilateral or bilateral), type of CSOM, and the stage of the disease at the time of presentation. An otoscopic evaluation of the ears was done to record the tympanic membrane findings. Subsequently, patients underwent pure tone audiometry to evaluate the type and degree of hearing loss.

Pure tone audiometry was conducted using the Maico MA42 audiometer (Maico Diagnostics, Minneapolis, MN, USA), covering frequencies of 250, 500, 1000, 2000, 4000, and 8000 Hz for both air and bone conduction thresholds. The audiometric average was derived from 500, 1000, and 2000 Hz frequencies. A threshold of less than 25 dB in the worst ear was considered as "no hearing impairment." According to the American Speech-Language-Hearing Association grading of hearing impairment, hearing loss was categorized into mild (26-40 dB), moderate (41-55 dB), moderately severe (56-70 dB), severe (71-90 dB) and profound (≥91 dB) [4].

All participants were interviewed with two pre-validated, self-administered questionnaires: DASS 21 and the CES questionnaire.

The DASS 21 was developed by Lovibond and Lovibond to evaluate three self-report scales designed for measuring negative emotional states related to depression, anxiety, and stress [5]. While multiple scales are available for assessing psychological well-being, the DASS 21 stands out for its superior factor loading separation compared to other measures. Its unique tripartite framework distinguishes between depression, anxiety, and stress, enabling a more focused evaluation of these interlinked psychological states. It comprises a set of three self-report scales for depression, anxiety, and stress. Each of these scales are composed of seven items, further organized into subscales with similar content. The reliability of the three scales is deemed satisfactory, with adequate test-retest reliability at 0.71 for depression, 0.79 for anxiety, and 0.81 for stress [6].

The CES is a validated measurement tool, designed to assess the QoL in patients with chronic ear disease [7]. It was introduced as a disease-specific 13-item Likert scale comprising three distinct categories: an activity restriction-based subscale (AR), a symptom subscale (ST), and a medical resource utilization (MR) subscale.

Both questionnaires were translated into Hindi, considering the proficiency of the study population in the Hindi language. Hindi translation was done by professional translators who also conducted forward and back translations. Based on the participant’s response, a score ranging from 0 to 100 was calculated, where a higher score indicated better health, while a lower score suggested poorer health.

All the data were recorded in Microsoft Excel (Microsoft Corporation, Redmond, WA) worksheets and statistical analyses were conducted using IBM SPSS Statistics for Windows, version 29.0 (IBM Corp., Armonk, NY). Categorical variables were presented as numbers and percentages, while continuous variables were expressed as means ± standard deviations (SDs). To test mean differences of activity restriction symptoms, medical resource utilization, and CES scores between genders and the laterality of the affected ear, an unpaired t-test was employed. Pearson’s correlation was applied to test the correlation between CSOM and QoL and psychosocial status. A P-value of <0.05 was considered statistically significant.

## Results

From the original cohort of 182 patients diagnosed with CSOM, 32 individuals were excluded from the study based on predefined exclusion criteria. The excluded participants comprised five patients with a history of road traffic accidents resulting in head injury, 22 patients with various systemic comorbidities, three patients with a history of depression, and two patients presenting with otogenic brain abscesses. These exclusions were essential to ensure that the study focused on a cohort with a final sample size of 150 patients without any confounding factors that could impact the assessment of disease-specific quality of life and psychological well-being associated with CSOM.

A total of 150 cases were included in this study. The age range was 18-72 years, with a mean age of 34.3 years (Table 2). The gender distribution revealed a predominance of females, constituting 63.3% (95 patients), while males accounted for 36.6% (55 patients) of those affected. In the study population with CSOM, 58.7% reported ear discharge for less than five years, while 32.0% had a 5- to 10-year duration and 9.3% experienced ear discharge for over 10 years. The distribution of ear involvement in the study cohort revealed that 63.3% of cases had unilateral CSOM, while 36.6% presented with bilateral CSOM. On otoscopic evaluation, 84 (56.0%) had a safe or mucosal type of CSOM, and 66(44.0%) were affected by the unsafe or squamosal type of CSOM. In terms of complications, 92.7% reported no complications, while 5.3% experienced mastoiditis. Facial palsy and labyrinthitis were observed in 1.3% and 0.7% of cases, respectively. On pure tone audiometry, 85 (56.7%) had mild hearing loss, 47 (31.3%) had moderate hearing loss, 15 (10.0%) had moderately severe hearing loss, and 3 (2.0%) patients had severe hearing loss.

**Table 2:** Demographic and clinical characteristics of the study population (n=150) CSOM: chronic suppurative otitis media

Characteristics	n	%
Gender		
Male	55	63.3
Female	95	36.6
Age (years)	34.3 (18.0-72.0)	
Duration of ear discharge		
<5 years	88	58.7
5-10 years	48	32.0
>10 years	14	9.3
Ear affected with CSOM		
Unilateral	95	63.4
Bilateral	55	36.6
Type of CSOM		
Safe CSOM	84	56.0
Unsafe CSOM	66	44.0
Complications of CSOM		
No complications	139	92.7
Mastoiditis	8	5.3
Facial palsy	2	1.3
Labyrinthitis	1	0.7
Degree of hearing loss		
Mild	85	56.7
Moderate	47	31.3
Moderately severe	15	10.0
Severe	3	2.0

**Figure 1:**
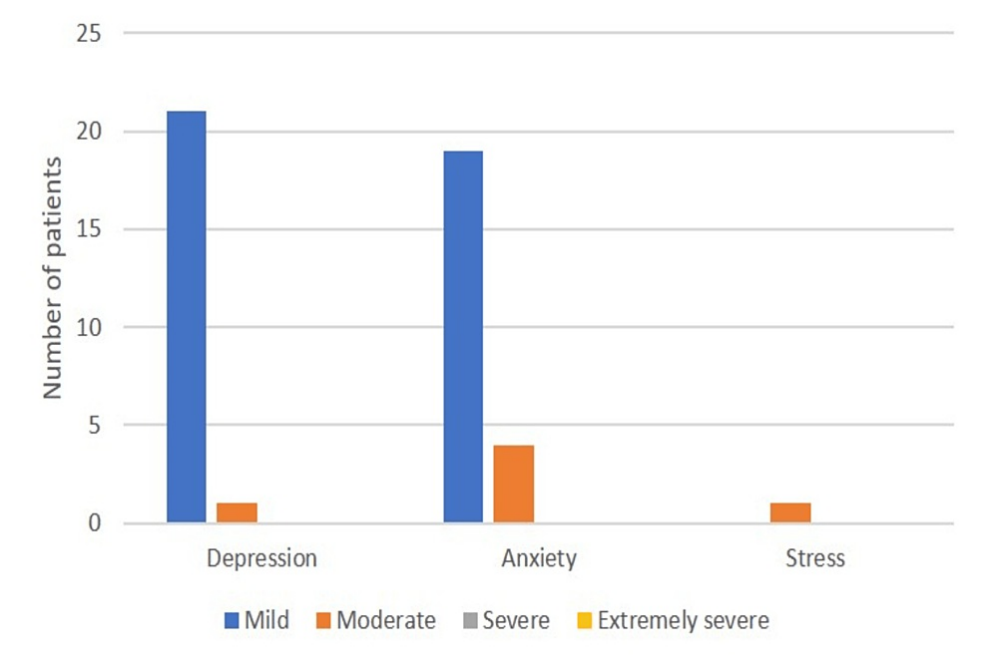
Distribution of patients according to psychological evaluation with the DASS 21 questionnaire The data are presented in the form of n (%). DASS 21: 21-item Depression, Anxiety, and Stress Scale

In the assessment of psychological well-being by the DASS 21 questionnaire, depression was observed in 22 (14.7%) patients, and anxiety was noted in 23 (15.3%) patients. Among those with depression, majority of the participants were having mild depression. Similarly, among those with anxiety, the majority were experiencing mild anxiety. Moderate stress was noted in only one CSOM patient who presented with facial palsy. None of the participants experienced severe or extremely severe depression, anxiety, or stress (Figure 1).

Among individuals with unilateral CSOM (n=95), 8 (8.4%) patients experienced anxiety, while 10 (10.5%) exhibited symptoms of depression. In the bilateral CSOM group (n=55), 15 (27.2%) individuals were found to have anxiety, and 12 (21.8%) had symptoms of depression. The statistical analysis demonstrated a significant association with laterality, particularly in the case of anxiety (Table 3).

**Table 3:** Association of anxiety and depression with laterality of the ear affected with CSOM The data are presented in the form of n (%). CSOM: chronic suppurative otitis media

	Ear affected with CSOM		Total	P value
	Unilateral (n=95)	Bilateral (n=55)		
Anxiety	8 (8.4)	15 (27.2)	23	0.002
Depression	10 (10.5)	12 (21.8)	22	0.060

The evaluation of QoL parameters using the CES questionnaire demonstrated significant associations with the laterality of ear involvement in CSOM patients. As depicted in Table 4, mean scores within each category of the CES questionnaire were analyzed. All the QoL parameters were found to be worse in the bilateral CSOM group as compared to the unilateral CSOM group. Statistically significant associations were observed in all categories except for symptoms. No significant association between gender and QoL parameters was observed among male and female participants.

**Table 4:** Association of QoL parameters in the CES questionnaire with laterality of the ear affected with chronic suppurative otitis media The data are presented in the form of means ± standard deviations. CES: chronic ear survey; QoL: quality of life

QoL parameters	Ear affected	Mean score obtained in the CES questionnaire	Mean difference	P value
Activity restriction	Unilateral	69.1±18.5	15.342	<0.001
	Bilateral	53.8±14.4		
Symptoms	Unilateral	75.0±15.2	5.624	0.017
	Bilateral	69.4±10.9		
Medical resource utilisation	Unilateral	75.9±11.3	7.025	<0.001
	Bilateral	68.9±11.4		
Total CES score	Unilateral	73.4±13.0	9.182	<0.001
	Bilateral	64.2±8.9		

Table 5 illustrates the correlation between psychological well-being (mean DASS 21 scores) and the quality of life (mean total CES scores) with the degree of hearing loss. For the DASS 21 scores, the mean scores increased with the severity of hearing loss, which was statistically significant (F value = 6.294, P<0.001). Likewise, a similar trend was observed for the total CES scores with increasing severity of hearing loss. Again, a statistically significant difference was noted (F value = 3.193, P<0.001). These results highlight the impact of the degree of hearing loss on psychological well-being and overall quality of life in individuals with chronic suppurative otitis media.

**Table 5:** Correlation of degree of hearing loss with the mean DASS 21 score and mean total CES score The data are presented in the form of means ± standard deviations. CES: chronic ear survey; DASS 21: 21-item Depression, Anxiety, and Stress Scale

Scores	Degree of hearing loss	N	Mean score obtained	F value	P value
DASS 21 score	Mild	85	1.74±2.95	6.294	<0.001
	Moderate	47	2.85±2.59		
	Moderately severe	15	3.33±3.67		
	Severe	3	7.33±1.15		
	Total	150	2.36±3.03		
Total CES score	Mild	85	75.04±12.89	3.193	<0.001
	Moderate	47	65.13±8.43		
	Moderately severe	15	59.13±6.50		
	Severe	3	59.67±2.08		
	Total	150	70.03±12.47		

## Discussion

Chronic suppurative otitis media is characterized by recurring purulent discharge, perforation of the tympanic membrane, and accompanying hearing loss. Individuals with CSOM, especially those with substantial hearing loss, may tend to withdraw from social engagements, influencing their overall quality of life. The persistence of CSOM symptoms, such as recurrent ear discharge, ear discomfort, and frequent clinic visits, can further exacerbate the impact on patients' QoL. These troublesome symptoms, often accompanied by social disengagement, anxiety, and sadness, contribute to a diminished QoL across various dimensions, encompassing physical, social, functional, psychological, and family aspects [8].

The study encompassed a sample consisting of adults aged between 18 and 72 years. Psychological well-being, assessed using the DASS 21 questionnaire, indicated depression in 22 (14.6%) patients and anxiety in 23 (15.3%) patients. Majority of patients experienced mild anxiety. Notably, only one patient with facial palsy exhibited moderate stress, and no severe or extremely severe depression, anxiety, or stress cases were identified. In a study conducted by Bakir et al. in patients with CSOM and hearing loss, it was observed that CSOM patients with mild or moderate hearing loss have a poorer life quality and higher psychological problems [8]. Another cross-sectional study by Kumar et al. analyzing depression in CSOM revealed a 30% prevalence of depression, with females being more commonly affected than males [9]. The majority of the patients in their study had mild depression. Similarly, a prospective study conducted by Mehboob et al. observed that female patients in their selected groups experienced higher levels of hearing loss and depression compared to male patients [10]. Conversely, our study findings revealed no correlation between gender and depression, anxiety, or stress.

While these studies have primarily explored the impact of hearing impairment on quality of life, limited attention has been devoted to other prevalent symptoms associated with CSOM, such as ear discharge from one or both ears. A discharging ear may contribute to an additional decline in psychological health and quality of life among CSOM patients. Our study findings highlight that both quality of life and psychological well-being are more adversely affected in the bilateral CSOM group compared to the unilateral CSOM group. A recent study, focusing on the long-term impact of a draining cavity in patients undergoing mastoid obliteration surgery, highlighted the importance of a discharging ear in an individual's quality of life [11].

Our study observed elevated anxiety levels in patients with bilateral CSOM in comparison to those with unilateral CSOM. This trend is consistent with findings reported in other studies [12,13]. As assessed by the CES questionnaire, QoL parameters demonstrated worse outcomes in the bilateral CSOM group across all categories. Patients with bilateral CSOM often experience heightened anxiety and a poorer quality of life compared to those with unilateral CSOM due to several interconnected factors. Bilateral involvement exacerbates the impact on auditory function, leading to increased concerns about communication difficulties and social interactions. The challenges in daily activities, coupled with the persistent nature of symptoms such as ear discharge and discomfort in both ears, contribute to elevated anxiety levels [14]. The cumulative burden of managing symptoms in both ears, potential complications, and the perceived impact on the overall well-being significantly diminishes the quality of life of individuals with bilateral CSOM [14].

In this study, we noted a significant correlation between psychological well-being and the quality of life changing with the severity of hearing loss. This finding aligns with a community-based cross-sectional study conducted by Contrera et al., which also reported increased anxiety levels in individuals with mild to moderate hearing impairment [15]. This can be attributed to the profound impact that hearing impairment can have on various aspects of an individual's life. As the severity of hearing loss increases, individuals may face greater challenges in effectively communicating with others, participating in social events, and maintaining a sense of connectedness. Feelings of isolation, frustration, and difficulty in engaging with one's surroundings may intensify with worsening hearing impairment [16]. Additionally, individuals with severe hearing loss may experience limitations in professional and personal relationships, potentially leading to increased stress, anxiety, and a sense of social withdrawal. Furthermore, the impact of hearing loss on one's ability to enjoy various aspects of life, such as music, conversations, and environmental sounds, can contribute to a reduced overall quality of life [17,18]. The interdependence of psychological well-being and quality of life in the context of severe hearing loss highlights the need for early psychological as well as surgical intervention.

Limitations

While this study provides valuable insights into the correlation between psychological well-being, quality of life, and hearing loss in individuals with chronic suppurative otitis media using validated instruments, it is essential to acknowledge certain limitations. The cross-sectional nature of the study restricts the ability to establish causation or intertemporal relationships. A longitudinal study could offer a more comprehensive understanding of the dynamic interplay between variables in the pre-operative as well as post-operative period. The study's sample may not fully represent the diversity of individuals with CSOM, as it is confined to the central Indian population only. Generalizing findings to broader populations should be done cautiously. The study relies on self-report measures for psychological well-being and quality of life. Despite conducting face-to-face interviews, it is important to acknowledge the potential for response bias in this study, as participants might have provided socially desirable answers or misinterpreted questions.

## Conclusions

Chronic suppurative otitis media significantly impacts individuals' lives, extending beyond auditory health. The findings of this study highlight a significant level of anxiety in CSOM patients with bilateral ear involvement and in patients with severe hearing loss. This association is likely linked to the profound impact of hearing loss and the severity of symptoms on their overall psychological well-being and quality of life. Recognizing these psychosocial challenges, clinicians treating individuals with CSOM should consider integrating psychosocial support or interventions into their care plans. Regular assessments of depression, anxiety, and stress, stemming from CSOM-related symptoms and activity restrictions, are essential during and after treatment. This holistic approach not only addresses the physical symptoms but also guides caregivers in supporting patients both mentally and physically.
